# Optimization Design of Laser Arrays Based on Absorption Spectroscopy Imaging for Detecting Temperature and Concentration Fields

**DOI:** 10.3390/ma17143569

**Published:** 2024-07-18

**Authors:** Limei Fan, Fangxu Dong, Jian Duan, Yan Sun, Fei Wang, Junyan Liu, Zhenhe Tang, Liangwen Sun

**Affiliations:** 1 Shandong Nonmetallic Materials Institute, Jinan 250031, China; limei8729@126.com (L.F.); duanjian_@163.com (J.D.); wosyh@163.com (Y.S.); jinan53tang@126.com (Z.T.); slw53nlyz@163.com (L.S.); 2 School of Mechatronics Engineering, Harbin Institute of Technology, Harbin 150001, China; wangfeipublic@163.com (F.W.); ljywlj@hit.edu.cn (J.L.)

**Keywords:** TDLAS technology, optical path optimization, high-temperature flame, chromatographic imaging

## Abstract

Detecting temperature and concentration fields within engine combustors holds paramount significance in enhancing combustion efficiency and ensuring operational safety. Within the realm of engine combustors, the laminar absorption spectroscopy technique has garnered considerable attention. Particularly crucial is the optimization of the optical path configuration to enhance the efficacy of reconstruction. This study presents a flame parameter field reconstruction model founded on laminar absorption spectroscopy. Furthermore, an optimization approach for refining the optical path configuration is delineated. In addressing non-axisymmetric flames, the simulated annealing algorithm (SA) and Harris’s Hawk algorithm (HHO) are employed to optimize the optical path layout across varying beam quantities. The findings underscore a marked reduction in imaging errors with the optimized optical path configuration compared to conventional setups, thereby elevating detection precision. Notably, the HHO algorithm demonstrates superior performance over the SA algorithm in terms of optimization outcomes and computational efficiency. Compared with the parallel optical path, the optimized optical path of the HHO algorithm reduces the temperature field error by 25.5% and the concentration field error by 26.5%.

## 1. Introduction

The detection and evaluation of combustion flow characteristics within an engine have become pivotal topics in contemporary aero-engine research. The detection of parameters such as temperature and concentration fields is crucial for combustion diagnostics. These parameters not only accurately reflect the state of flame combustion but also provide essential indicators for assessing combustion quality. By analyzing and monitoring these parameters, researchers can effectively evaluate flame combustion efficiency, completeness, and potential emission levels. This process provides essential foundations for optimizing combustion processes and controlling pollution. Accurately and swiftly measuring the temperature and concentration fields within the combustion chamber is critical across aerospace, thermal power generation, environmental monitoring, and related fields [1,2,3,4]. Tunable Diode Laser Absorption Spectroscopy (TDLAS) stands out as a non-contact diagnostic technique with extensive applications in environmental monitoring [5,6,7], trace analysis [8,9,10], combustion diagnostics [11], plasma diagnostics [12], and beyond. Originating in the last century, TDLAS principles and methods have seen widespread adoption in the current century. The rapid advancement of TDLAS technology can be attributed to several key factors: developments in optical communication and photonics, the continual enhancement of spectral databases, and advancements in testing technologies. Innovations in optical communication and optoelectronics have addressed challenges related to laser sources and detection, facilitating reliable multi-laser beam combining and decomposition. Meanwhile, improved spectral databases offer robust tools for selecting absorption lines and inverting spectral data. Progress in testing technology has resolved issues concerning multi-channel, high-precision data acquisition, and real-time processing. Additionally, TDLAS technology boasts advantages such as a compact structure, rapid data processing capabilities, and the ability to perform simultaneous online detection of multiple variables. These attributes further underscore the significance and versatility of TDLAS in various scientific and industrial applications.

During the combustion process, spatial constraints within the combustion device limit possible beam arrangements and impose restrictions on optical access. Given these limitations in spatial sampling, optimizing beam arrangement becomes crucial for achieving precise reconstruction [13,14,15]. In two-dimensional gas distribution reconstruction, the quality of results is directly influenced by factors such as projection angle and the number of rays used with parallel or fan beams. Practical combustion installations face space constraints, making it challenging to install numerous optical sensors. To enhance performance and minimize the number of sensors in a combustion system, researchers must carefully select the optimal sensor distribution for tomographic reconstruction. While a fan-shaped beam arrangement reduces emitter costs, it distorts the corners of the reconstructed image. In contrast, irregular beam arrangements employ optimization strategies to reduce the number of beams, thereby significantly improving measurement accuracy.

Matthew et al. devised a novel optical path arrangement algorithm to probe the correlation between the mathematical properties of the coefficient matrix and the information content of the attenuation data. This laid the foundation for a beam arrangement design algorithm that minimizes reliance on additional assumed information regarding concentration distribution. When tested in simulated laser absorption tomography experiments, the optimized beam arrangement exhibited superior reconstruction accuracy compared to other configurations in the literature [16]. Song et al. delved into the interplay between the grid count and projected ray count when employing parallel and sector beams for edgewise projection. They introduced the virtual light method, which significantly enhances reconstruction quality. Furthermore, they explored an optimization strategy for ray distribution in fan beam projection [17]. Xin et al. addressed the rank-loss issue in tomographic reconstruction for combustion diagnosis by adjusting the weighting of the conditioning equation based on the number of rays traversing the grid cells. They proposed a novel regularization technique considering neighboring grid values and ray counts passing through the grid cells. Numerical simulations demonstrated that this new adjustment method alleviates reconstruction errors and effectively suppresses angular distortion in the four projection angles. The impact of weighting coefficients and smoothing factors on the reconstruction was scrutinized via numerical investigations. Combustion experiments validated that the new conditioning method reduces reconstruction errors, particularly under non-ray crossing conditions [18]. While the aforementioned researchers explored optical path arrangement methods to enhance temperature detection quality, none of their strategies were deemed optimal, necessitating further optimization. Hence, this study employs the simulated annealing algorithm and the Harris’s Hawk algorithm to delve deeper into optimizing the optical path arrangement.

Combustion diagnostic methodologies involve addressing the inverse problem, using observational data to infer the parameters governing the physical system. This inverse challenge inherently poses an optimization dilemma. In practical applications such as internal combustion engines and gas turbines, spatial constraints limit the feasible array of beam configurations. To enhance combustion system efficiency and optimize sensor deployment, selecting an optimal sensor layout in tomographic reconstructions is crucial for improving temperature imaging precision. This study introduces a temperature field reconstruction methodology based on Tunable Diode Laser Absorption Spectroscopy (TDLAS). The optical path arrangement is then optimized using simulated annealing and the Harris’s Hawk algorithm.

## 2. Methods

### 2.1. Linear Tomography Flame Imaging Measurement Model

**Figure 1:**
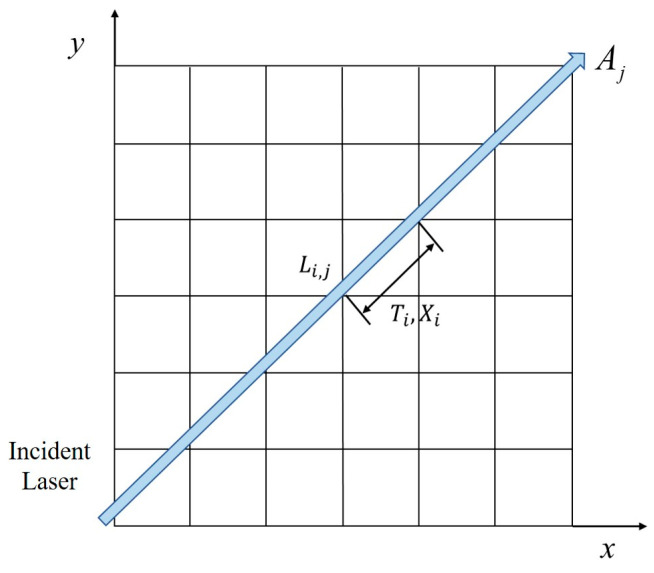
Schematic diagram of the measurement area.

The Tunable Diode Laser Absorption Tomography (TDLAT) technique, which integrates Tunable Diode Laser Absorption Spectroscopy (TDLAS) with Computed Tomography (CT), serves as the foundational approach in this study. By addressing the inverse problem using the measured signals obtained from spectral data collected along multiple optical paths, we can derive the temperature and concentration distributions within the measured region in two dimensions, as depicted in Figure 1. Employing the apparent light method along each ray, the integral absorbance of the entire measured spectral line can be expressed in a discretized form.

(1) $$A_{v,j}=\sum_{j=1}^{N}{\left[PS(T)X\right]}_{v,j}L_{i,j}=\sum_{j=1}^{N}f_{v,j}L_{i,j}$$

where *A_v,j_* represents the integrated absorbance obtained by the j laser ray with the frequency of v and *N* is the total number of grid points (*j* = 1,2,…, *N*, *N* = *n*^2^). Following the principles of tomography, the absorption coefficients *f_v,j_* for each grid point are computed at the wavelengths corresponding to the two independent spectral lines, incorporating the spectral line intensities *S_v,j_*(*T*), the gas of the substance to be measured *X* mole fraction, and the total pressure parameter *P*, thus inferring the gas temperature and concentration of the component to be measured. *L_i,j_* is the length of the ray *i* through the grid point *j*, but this length is only related to the angle and position.

When the total number of projected rays is *M*, it can be expressed as follows:

(2) $$\left\{\begin{array}{c} L_{11}f_{1}+L_{12}f_{2}+\ldots +L_{1N}f_{N}=A_{1}\\ L_{21}f_{1}+L_{22}f_{2}+\ldots +L_{2N}f_{N}=A_{2}\\ \vdots \\ L_{M1}f_{1}+L_{M2}f_{2}+\ldots +L_{MN}f_{N}=A_{M} \end{array}\right.$$

In the formula, *A_M_* represents the integral absorbance matrix obtained from the measurement of *M* beams after passing through the area to be measured, and *M* is the dimension of the matrix; *L_MN_* represents the projection coefficient matrix of the area to be measured, and its rows and columns represent the number of grid divisions and the number of detection beams, respectively, and its value can be obtained by the relative relationship between the laser diode and the detection device during the experimental process; *f_N_* is the absorbance coefficient matrix, which is the physical quantity to be solved.

After calculating the absorbance coefficient matrix, if the temperature of the field to be measured is known, the concentration of the area to be measured can be calculated. A two-dimensional temperature-concentration measurement of the area to be measured is achieved.

In practical scenarios, the temperature and concentration distribution within the measured medium are frequently unknown, rendering it a challenging nonlinear inverse problem that defies direct resolution. To facilitate the concurrent measurement of temperature and component concentration, we integrate the ‘two-line ratio method’ with distributed optical fiber sensing technology, thereby establishing a system of equations for two distinct absorption spectral lines.

(3) $$\left\{\begin{array}{l} A_{J,V_{1}}=f_{J,V_{1}}L_{JV_{1}}\\ A_{J,V_{2}}=f_{J,V_{2}}L_{J,V_{2}} \end{array}\right.$$

The physical meaning of the line intensity function *S_i_*(*T*) is the description of the absorption strength of the absorbing jump line to a specific frequency ν of laser light under certain conditions: the line intensity function varies with the temperature, which can be expressed by the following equation [19]:

(4) $$S_{i}(T)=S\left(T_{0}\right)\frac{Q\left(T_{0}\right)}{Q(T)}\frac{T_{0}}{T}\exp\left[-\frac{hcE^{\prime \prime }}{k}\left(\frac{1}{T}-\frac{1}{T_{0}}\right)\right]\frac{1-\exp\left(-\frac{hcv_{0}}{kT}\right)}{1-\exp\left(-\frac{hcv_{0}}{kT_{0}}\right)}$$

The temperature can be deduced from the ratio of the integral absorbances of the two different spectral lines measured. Since the two integral absorbances are obtained at the same pressure path length and component concentration, the ratio of the two integrals can be easily simplified to a ratio of the line intensities [20,21].

(5) $$R(T)=\frac{A_{1}}{A_{2}}=\frac{\int PLXS_{1}(T)\Phi_{v_{1}}dv}{\int PLXS_{2}(T)\Phi_{v_{2}}dv}=\frac{S_{1}\left(T_{0}\right)}{S_{2}\left(T_{0}\right)}\exp\left[-\frac{hc}{k}\left(E_{2}^{\prime \prime }-E_{1}^{\prime \prime }\right)\left(\frac{1}{T}-\frac{1}{T_{0}}\right)\right]$$

The measured component temperature *T* can be obtained from the two low-state jump energy different absorption spectra, the temperature *T* relation is as follows.

(6) $$T=\frac{\frac{hc}{k}\left(E_{2}^{\prime \prime }-E_{1}^{\prime \prime }\right)}{\ln\left[\frac{A_{1}}{A_{2}}\right]+\ln\left[\frac{S_{2}\left(T_{0}\right)}{S_{1}\left(T_{0}\right)}\right]+\frac{hc}{k}\frac{\left(E_{2}^{\prime \prime }-E_{1}^{\prime \prime }\right)}{T_{0}}}$$

After the measurement of the component temperature *T*, the component concentration can be calculated from the available data as follows.

(7) $$X_{i}=\frac{A_{1}}{PS_{v_{1}}(T)L}$$

### 2.2. Optical Path Optimization Model

#### 2.2.1. Simulated Annealing Algorithm

**Figure 2:**
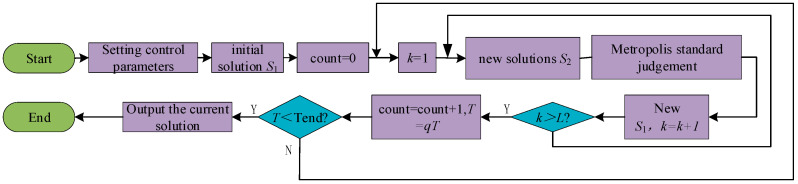
Simulated annealing algorithm solution flowchart. (Y: yes, N: no).

The simulated annealing algorithm (SA) was initially proposed by Metropolis, drawing inspiration from the annealing process of solid materials and addressing general combinatorial optimization problems. This versatile optimization algorithm adopts an iterative solution strategy akin to the Monte Carlo algorithm, introducing a probability for the solution process to escape locally optimal solutions and converge on globally optimal solutions. Figure 2 is the flow chart of the simulated annealing algorithm. Firstly, the model hyperparameters and initialization are set and then the iterative process is used to solve the problem. The physical annealing process consists of three key steps:

(1) Heating step: This phase intensifies the thermal movement of particles, disrupting the system’s equilibrium. With a sufficiently high temperature, the solid transitions into a liquid state, effectively eliminating the original system inhomogeneities.
(2) Isothermal step: At a constant temperature, the system’s state evolves in response to free energy, ultimately reaching equilibrium at the minimum free energy value.
(3) Cooling step: This phase decelerates the thermal motion of particles, reducing the system’s energy and yielding a crystalline structure.

When a simulated annealing algorithm is used, the main control parameters such as cooling rate *q*, initial temperature *T*_0_, terminal temperature *T*_end,_ and chain length *L* need to be set. Its control core is the Metropolis criterion: if the objective optimization function is *f* (*S*), then the result of the current solution is *f* (*S*_1_), the result of the new solution is *f* (*S*_2_), the difference of the result is *df* = *f* (*S*_2_) − *f* (*S*_1_), then the Metropolis criterion is as follows:

(8) $$P=\left\{\begin{array}{l} 1,\cdots \cdots \cdot d\cdot df<0\\ \exp\left(-\frac{df}{T}\right),\cdots df\geq 0 \end{array}\right.$$

If *df* < 0, then the new result is accepted with probability 1; otherwise, the new result is accepted. The cooling is performed using the cooling rate *q*, which is *T*= *qT*, and if *T* is less than the terminal temperature then the iteration is stopped and the current state is output; otherwise, the iteration continues. In CT-TDLAS 2D reconstruction solving, the simulated annealing algorithm is solved directly with the temperature field *T* and the concentration field *X* as the unknown quantities, hence its name as a nonlinear solving method.

#### 2.2.2. Harris’s Hawk Optimization Algorithm

Harris’s Hawks Optimization (HHO) is a population optimization algorithm proposed by Aaha et al., which simulates the predatory behavior of the Harris’s Hawk (a raptor in southern Arizona, USA), and is divided into an exploration phase, an exploration-exploitation conversion phase, and an exploitation phase [22].

(1) Exploration phase

The Harris’s Hawk relies on its keen eyesight to track and spot prey, although there are instances where the prey remains elusive. Consequently, the hawk dedicates hours to waiting, observing, and monitoring the desert landscape. Within the algorithmic framework, the Harris’s Hawk is conceptualized across various scenarios, with the iterative search for prey representing the optimal or near-optimal strategy for each state. These hawks establish random perches in trees within their territory and employ two strategic choices based on their discernment. When the probabilities (*q*) associated with each perching strategy are equal, the Harris’s Hawk selects a perch considering the positions of other members and the prey for *q* < 0.5. Conversely, for *q* > 0.5, the hawk randomly opts for a sizable tree as its perch, as modeled in the algorithm.

(9) $$X(t+1)=\left\{\begin{array}{cc} X_{rand}(t)-r_{1}\left|X_{rand}(t)-2r_{2}X(t)\right| & q\geq 0.5\\ \left(X_{rabbit}(t)-X_{m}(t)\right)-r_{3}\left(LB+r_{4}(UB-LB)\right) & q<0.5 \end{array}\right.$$

where *X*(*t* + 1) is the location of the Harris’s Hawk in the iterative process to be carried out, *X_rabbit_*(*t*) represents the optimal solution location (i.e., the location where the optimal solution is located) in the current state, *X(t)* is the location of the Harris’s Hawk in the current iteration, *r1, r2, r3, r4*, and *q* are the random numbers in (0, 1), *LB* and *UB* represent the algorithm’s unknowns of the demand solution of the upper and lower boundaries, *X_rand_* is the randomly chosen location of the Harris’s Hawk during this iteration, and *X_m_*(*t*) is the average amount of the location of the Harris’s Hawk in the iteration.

(10) $$X_{m}(t)=\frac{1}{N}\sum_{i=1}^{N}X_{i}(t)$$

(2) Transition from exploration to exploitation

The HHO algorithm shifts from ‘exploration’ to ‘exploitation’ and switches from ‘exploration’ to ‘exploitation’ according to the size of the escape energy of the ‘prey’. The HHO algorithm switches from ‘exploration’ to ‘exploitation’, and switches from ‘exploitation’ to ‘exploitation’ according to the amount of escape energy. When the prey escapes, their energy is greatly reduced. The exiting energy of the prey is calculated as follows:

(11) $$E=2E_{0}\left(1-\frac{t}{T}\right)$$

where *E* denotes the exergy of the predator (rabbit), *T* is the maximum number of iterations required for the iterative process, and *E*_0_ is the energy that the predator initially has.

The initial energy is random in the iterative process and its range is within (−1, 1). When the value of the initial energy decreases from 0 to −1, the prey records its current physical position, while when the value of the initial energy increases from 0 to 1, the rabbit starts to strengthen its active state. Overall, the exsanguination energy decreases during the iterative process. When the exergy $\left|E\right|\geq 1$, the Harris’s Hawk performs a global search to determine the coordinates where the rabbit is located, and therefore, the algorithm enters the exploration phase at this point; when $\left|E\right|<1$, the algorithm tries to find the neighborhood of the solution in the exploration phase. In summary, |E|≥1 performs exploration and exploitation (exploitation).

(3) Exploitation stage

In this stage, the predator attacks the target prey identified in the previous stage. However, the prey usually manages to escape. Therefore, different types of chasing behavior may arise in real-life environments. Based on the different predation strategies of Harris’s hawks and the behavioral patterns of rabbits, four possible strategies for simulating the predation phase are proposed.

The prey always wants to escape from danger. Let r be the chance of the prey escaping before a sudden attack, and the chances of both success and failure are split in half; when *r* = 0.5, it represents a failure to escape. Regardless of what the prey does, Harris’s Hawks always catch it in a strong or gently encircling manner. This means that they will gently or aggressively encircle their prey from different directions, depending on the physical strength of the prey. Harris’s Hawks are smart in conducting the hunt, gradually approaching the prey’s position based on the judgment of the existing situation and echoing with their companions to kill the prey together; then, Harris’s Hawks intensify the encirclement so that the exhausted prey can be easily captured. The parameter *E* is defined to model this strategy, in a model that enables the algorithm to emulate the Harris’s Hawk’s choice of soft or hard siege depending on the situation. When *E* ≥ 0.5, the soft siege is performed; when *E* < 0.5, the hard siege is performed.

The primary advantage of the Harris’s Hawks Optimization (HHO) algorithm is its independence from external parameters during the optimization process, allowing it to accurately identify the optimal solution for the objective function. The steps for optical path optimization based on the HHO algorithm are as follows:

(1) Initialization: Define initial parameters such as population size, number of iterations, and the objective function. Randomly generate variables and calculate the objective value of the initial solution. Concurrently, iterates the algorithm by generating random variables again to update the global optimal solution, storing iteration data for subsequent comparison.
(2) Exploration: Update the random light path coordinate positions according to the algorithm’s rules and perturb these coordinates based on the computation’s scale.
(3) Exploitation: perform hard and soft sieges sequentially, updating the coordinate positions and target values in each iteration according to the computational formulas.
(4) Progressive Fast Swooping Siege Strategy: Update the target value and corresponding coordinates as per the formula. At the end of each iteration, update the global best solution, recording the best solution for each generation. Repeat the iterations for the specified number of times to ultimately obtain the optimal optical path and the highest reconstruction accuracy.

For the iteration of the whole model, which in this simulation is essentially a regression problem in the optimization model problem, the loss function *L* is as follows:

(12) $$L=\mathrm{MSE}=\frac{1}{N}\sum_{i=1}^{N}{\left(T_{ri}-T_{mi}\right)}^{2}$$

where *N* is the total number of grids after the one-dimensional unfolding of a single temperature matrix, the temperature is calculated at each iteration of the *T_m_* optimization algorithm, and *T_r_* is the actual set temperature field.

## 3. Results and Discussion

**Figure 3:**
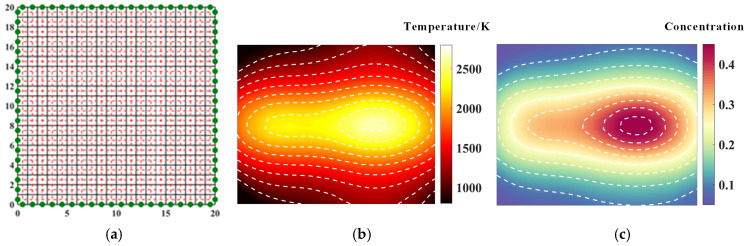
Reconstruction of temperature field and concentration field under regular optical path: (**a**) optical path arrangement, 40 beams, (red dotted line: light path, black solid line: flame field grid); (**b**) temperature field; (**c**) concentration field.

In this paper, the linear optical flame imaging model established in the first section is used to reconstruct the flame parameter field. The calculation results are based on numerical simulation and will be extended to experimental measurement in subsequent applications. As shown in Figure 3, the parameter field reconstruction is performed using a 40-rule parallel light path. The flame temperature and concentration field measured by the parallel light path can reflect the shape of the flame compared with the random light path. The average relative error of the temperature under the parallel light path is 0.051, and the average relative error of the concentration is 0.083. The results can be compared with the optimized random light path measurement results.

In practical measurement scenarios, the subject is often a multimodal flame. Therefore, during the process of optical path optimization, it is essential to prioritize a multimodal bimodal flame as the target for optimizing the optical path layout. Furthermore, the parameters of the optimization algorithm should be carefully considered. Table 1 below illustrates the application of the simulated annealing algorithm for optimizing the optical path layout:

With this parameter configuration, one round of optimization is performed for the randomly generated light path, with 10 iterations conducted at each temperature. After 44 iterations, the temperature converges and the optimization process is terminated, selecting the flame morphology as a multimodal flame. For the HHO optimization algorithm, the number of iterations is set to 500, and the population size is 20. During the entire iteration process of the light path optimization algorithm, the root mean square difference error, as shown in the following equation, is used as an evaluation metric.

(13) $$\mathrm{MSE}=\frac{1}{N}\sum_{i=1}^{N}{\left(T_{ri}-T_{mi}\right)}^{2}$$

where *N* is the temperature on each grid for the temperature distribution function used, *T_mi_* is the temperature obtained at each iteration for the different optical paths in the algorithm, and *T_ri_* is the actual set temperature distribution.

### 3.1. Optimization of the Optical Path for Symmetric Measurements

**Figure 4:**
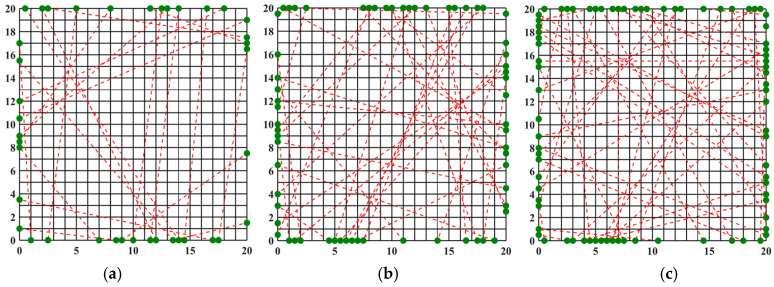
SA beam arrangement obtained after optimization: (**a**) 20 beams; (**b**) 32 beams; (**c**) 40 beams, (red dotted line: light path, black solid line: flame field grid).

An asymmetric measurement light path is characterized by the emission of beams from all four symmetric edges during light path generation. The optimization results and the finest optical path arrangement achieved through the simulated annealing algorithm under 20, 32, and 40 optical path arrangements are depicted in Figure 4. The light path diagram following the optimization by the simulated annealing algorithm is presented in Figure 3, demonstrating a more uniform distribution of random light paths across the grid region and a significantly enhanced reconstruction effect compared to the unoptimized random light paths. Observing Figure 4, it becomes evident that the number of randomly arranged lights has a discernible impact on the reconstruction effect. Employing 20 random lights for the light path arrangement to measure the flame temperature field inadequately reflects the morphology of the multimodal flame. Conversely, utilizing 32 and 40 lights yields improved reconstructions of the flame temperature field, providing a clearer representation of the flame image. It is noteworthy that for the two-dimensional plane, the reconstruction of the multimodal flame using 32 beams excels in capturing the temperature peak with a higher amplitude; however, it falters in adequately reconstructing the other peak, which is relatively smaller in the two-dimensional plane, protruding only at the tip. On the other hand, the reconstruction of the multimodal flame using 40 beams is clear and delivers the optimal reconstruction effect among the three different quantities of light beams.

As shown in Table 2, the average relative error of the random light path arrangement using 32 beams and 40 beams is smaller than that of the 20-beam arrangement. Additionally, the error in the flame temperature field reconstructed with 32 and 40 beams is smaller than that obtained from the 40-beam parallel light path arrangement, suggesting that the temperature field reconstruction meets quality requirements. However, the reconstructed concentration field exhibits a relatively large error, and increasing the number of beams does not significantly improve the accuracy of the concentration field. Overall, the temperature reconstruction is more accurate than the concentration reconstruction. This discrepancy may be attributed to the weaker absorption lines at lower temperatures, which affect the inversion of concentration due to measurement errors in the boundary layer. In contrast, temperature inversion benefits from using the intensity ratio of two absorption lines to mitigate noise introduced by these lines.

**Figure 5:**
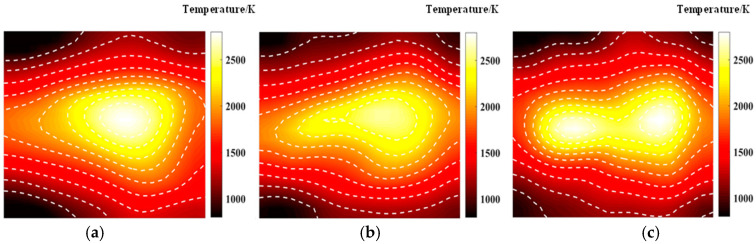
Reconstructed flame temperature obtained after SA optimization: (**a**) 20 beams; (**b**) 32 beams; (**c**) 40 beams.

**Figure 6:**
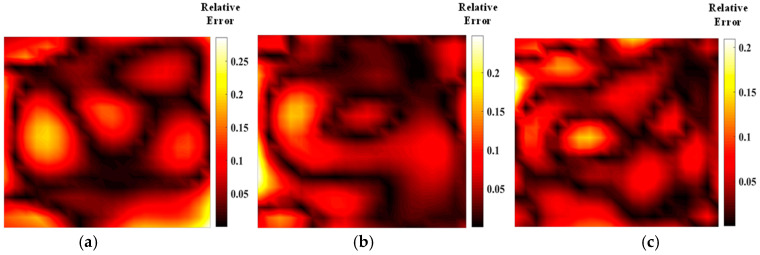
Relative error of reconstructed flame temperature obtained after SA optimization: (**a**) 20 beams; (**b**) 32 beams; (**c**) 40 beams.

**Figure 7:**
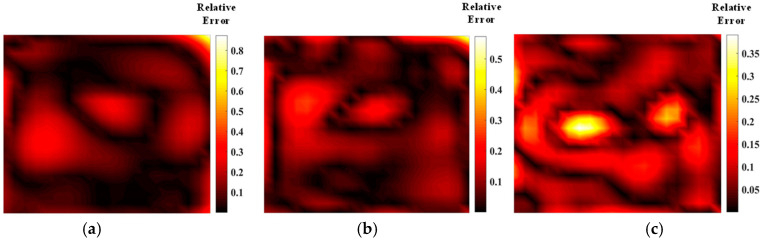
Relative error of reconstructed flame concentration obtained after SA optimization: (**a**) 20 beams; (**b**) 32 beams; (**c**) 40 beams.

Figure 5 shows the temperature field of the optical path arrangement after reconstruction and optimization by a simulated annealing algorithm. The temperature field is between 1000 K and 2500 K, and the white dotted line in the figure is the isotherm. The number of light paths used is 20, 30, and 40, respectively. The findings from Figure 6 and Figure 7 indicate that the reconstruction of concentration using 32 and 40 beams surpasses that obtained with 20 light rays. The relative error in reconstructing the flame concentration field is primarily concentrated at the peak of the multimodal flame, with higher reconstruction quality observed in the flame’s low-temperature and root regions. Conversely, a larger measurement error is concentrated in the edge region for the concentration field measured by the 40-beam arrangement, and a substantial measurement error occurs in the center region of the image. This discrepancy is attributed to the sparse distribution of light beams, with some grids remaining unexposed, leading to increased measurement errors. In contrast, the overall reconstruction of the temperature field demonstrates higher quality, with errors primarily concentrated at the edge region and in regions with sparse light coverage.

**Figure 8:**
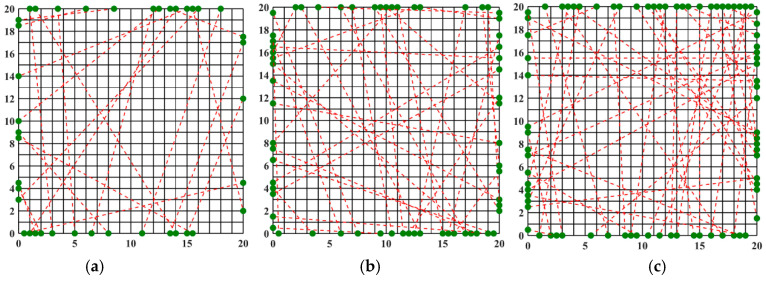
Beam arrangement obtained after HHO optimization: (**a**) 20 beams; (**b**) 32 beams; (**c**) 40 beams (red dotted line: light path, black solid line: flame field grid).

The optimized light path arrangement using the simulated annealing algorithm effectively enhances the reconstruction effect. However, further investigation is required to determine whether the reconstruction effect can be improved by adjusting the algorithm’s parameters or by employing a new optimization algorithm. Given the extensive prior research on the simulated annealing algorithm and light path optimization, a new optimization algorithm was employed to achieve the optimal arrangement of random light paths. The algorithm’s parameters were configured as described above, with 500 iterations and a population size of 20 for computation, resulting in the optimized light path arrangement depicted in Figure 8.

By comparing the beam arrangements obtained through the optimization of the Harris’s Hawks Optimization (HHO) algorithm with those derived from the Simulated Annealing (SA) algorithm, we observe that for the 20-beam arrangements, the distribution and grid coverage of light paths are relatively similar between the two methods. However, in the cases of the 32- and 40-beam arrangements, the random light path arrangement optimized by the HHO algorithm does not exhibit increased sparsity. Instead, it demonstrates a more homogeneous grid coverage by light paths. To verify the quality of the reconstructed temperature and concentration distributions in the flow field, further analysis and validation using additional information are necessary. Based on these three light path arrangements, the two-dimensional temperature and concentration fields of a non-axisymmetric flame are reconstructed for comparative analysis.

**Figure 9:**
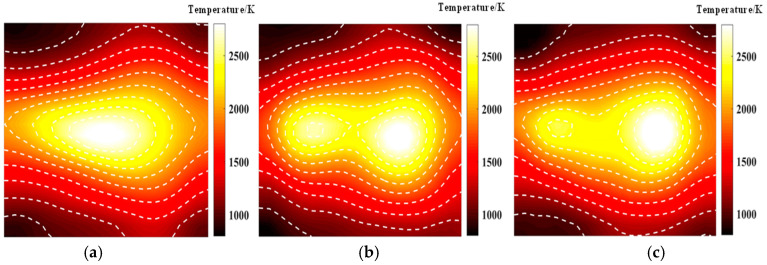
Reconstructed flame temperatures obtained after HHO optimization: (**a**) 20 beams; (**b**) 32 beams; (**c**) 40 beams.

**Figure 10:**
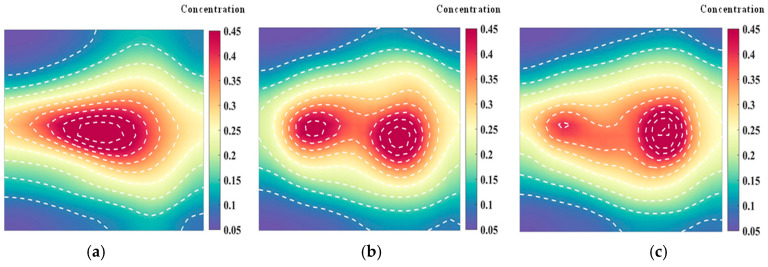
Reconstructed flame concentrations obtained after HHO optimization: (**a**) 20 beams; (**b**) 32 beams; (**c**) 40 beams.

Figure 9 and Figure 10 illustrate that similar to the results obtained with the simulated annealing algorithm, the reconstruction of temperature and concentration fields using a 20-beam arrangement is of poor quality. This arrangement fails to accurately capture the flame morphology, and the measurement results for both fields are consistent in their inadequacy. In contrast, when using the HHO algorithm with 32 beams, there is a significant improvement in measurement accuracy compared to the simulated annealing algorithm. The reconstructed images of the temperature and concentration fields more accurately reflect the flame distribution pattern. The reconstruction quality further improves with the optimized 40-beam arrangement, though the associated errors require additional discussion and analysis. Overall, the HHO algorithm substantially enhances the measurement accuracy of random light paths generated by 32 beams compared to the simulated annealing algorithm. This improvement provides valuable insights for optimizing light path arrangements in confined spaces.

**Figure 11:**
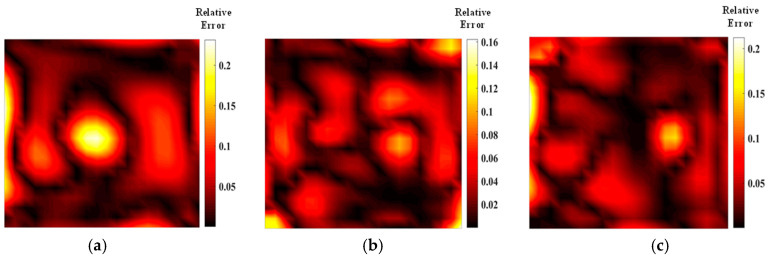
Relative error of reconstructed flame temperature obtained after HHO optimization: (**a**) 20 beams; (**b**) 32 beams; (**c**) 40 beams.

**Figure 12:**
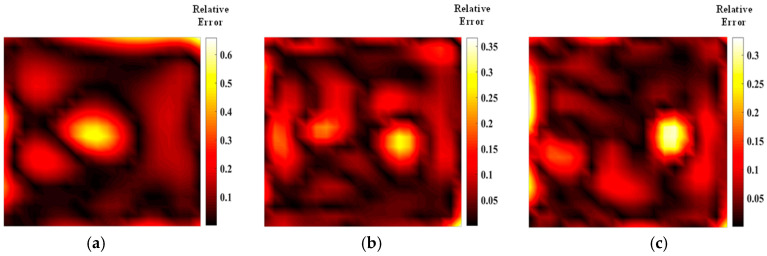
Relative errors of reconstructed concentrations obtained after HHO optimization: (**a**) 20 beams; (**b**) 32 beams; (**c**) 40 beams.

As shown in Figure 11 and Figure 12, the quality of the optimized optical path reconstruction for the flame temperature field is significantly enhanced, but the multimodal flame morphology is not well represented when using only 20 light rays. This is attributed to a large number of grid cells not being intersected by the laser beams. The reconstruction accuracy can be substantially improved by increasing the number of light rays. Notably, the average relative error of the temperature field obtained using the HHO algorithm with 32 light rays is 0.005 smaller than that achieved with a parallel optical arrangement of 40 light rays. Furthermore, increasing the number of random light rays to 40 does not result in a significant improvement in reconstruction quality, suggesting that image quality plateaus once the number of light rays reaches 32. The HHO algorithm demonstrates superior imaging quality and speed compared to the SA algorithm, confirming the feasibility and effectiveness of this novel optimization approach. Additionally, larger measurement errors are associated with the sparse arrangement of the optical paths, indicating that imaging quality can be further enhanced by adjusting the measurement area size and beam distribution. As shown in Table 3 and Table 4, the results of parameter field reconstruction using the HHO algorithm to optimize the optical path are better than those of the SA algorithm. Compared with the parallel optical path, the optimized optical path of the HHO algorithm reduces the temperature field error by 25.5% and the concentration field error by 26.5%.

### 3.2. Optimization of the Optical Path for Asymmetric Measurements

**Figure 13:**
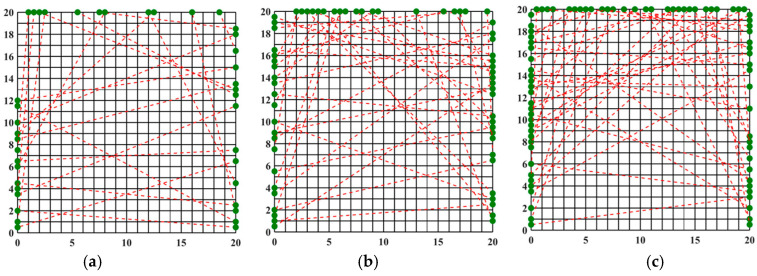
Asymmetric beam arrangement obtained after SA optimization: (**a**) 20 beams; (**b**) 32 beams; (**c**) 40 beams (red dotted line: light path, black solid line: flame field grid).

**Figure 14:**
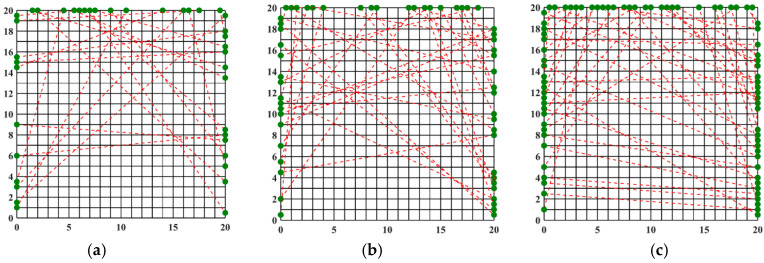
Asymmetric beam arrangement obtained after HHO optimization: (**a**) 20 beams; (**b**) 32 beams; (**c**) 40 beams (red dotted line: light path, black solid line: flame field grid).

In practical measurements, symmetrical arrangements are often limited, necessitating the use of asymmetrical configurations. The random light path for asymmetric measurements was optimized, employing a three-edge arrangement of lasers and detectors. The optimized asymmetric random light path arrangements for the two algorithms are depicted in Figure 13 and Figure 14. It is evident that the simulated annealing algorithm produces more uniformly distributed asymmetric random light paths with 20 and 32 beams. During the iterative process, to enhance reconstruction quality, the dominant side tends to be incorporated into the flame measurement region, whereas the weaker side, which likely corresponds to areas with less flame distribution, has minimal impact on improving measurement accuracy despite having more light paths arranged there. When the beam number is increased to 40, the light path arrangement begins to become more uniform as the number of light rays increases. However, the light paths optimized by the HHO algorithm still tend to have more beams concentrated on the weaker side of the measurement. This trend indicates that even with a higher number of beams, the distribution remains asymmetric, especially on the weaker side.

**Figure 15:**
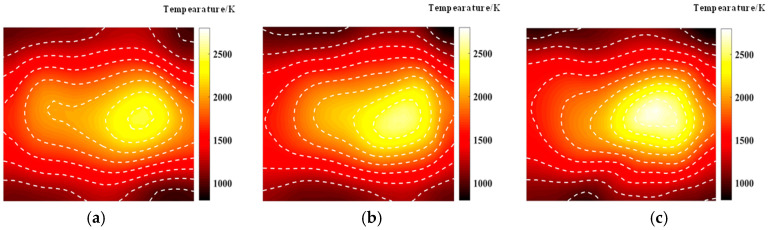
Reconstructed flame temperatures obtained after SA optimization of the asymmetric optical paths: (**a**) 20 beams; (**b**) 32 beams; (**c**) 40 beams.

**Figure 16:**
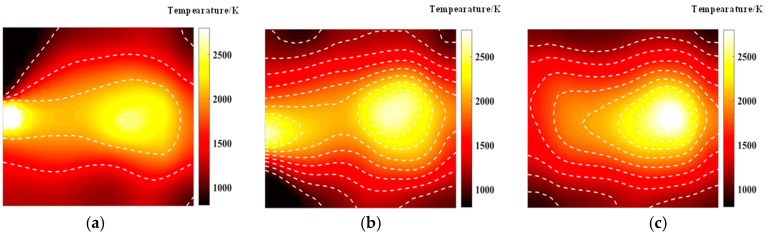
Reconstructed flame temperatures obtained after HHO optimization of the asymmetric optical circuit: (**a**) 20 beams; (**b**) 32 beams; (**c**) 40 beams.

The asymmetric distribution of light paths for flame measurement accuracy needs to be analyzed according to the specific flame distribution. Figure 15 and Figure 16 illustrate the asymmetric random light path reconstruction of the flame temperature and concentration fields, optimized by two different algorithms. The optimized light path arrangements for measuring the bimodal temperature field reveal that the reconstructed temperature field aligns more closely with the structure of the original flame field. In the case of the bimodal temperature field, one peak is measured more accurately, while the other peak remains inadequately captured. Despite the optimized asymmetric light path layouts showing gaps on the weaker side, these gaps result in artifacts during actual measurements, which do not correspond to the true structure of the flame temperature field. Nevertheless, the optimized asymmetric light paths developed by both algorithms demonstrate superior measurement results on the dominant side.

**Figure 17:**
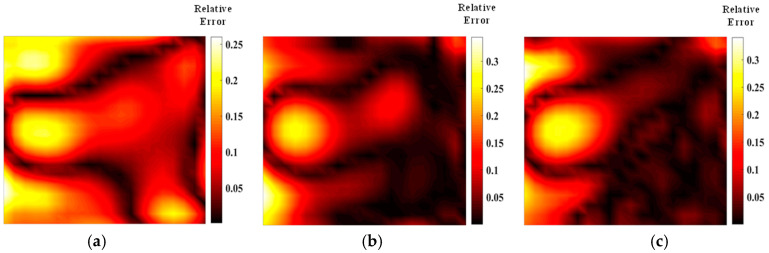
SA optimized asymmetric optical path temperature mean error distribution: (**a**) 20 beams; (**b**) 32 beams; (**c**) 40 beams.

**Figure 18:**
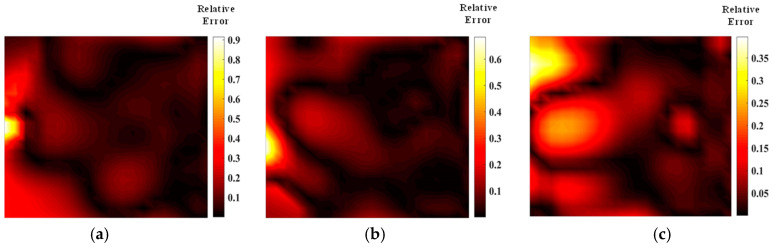
HHO optimized asymmetric optical path temperature mean error distribution: (**a**) 20 beams; (**b**) 32 beams; (**c**) 40 beams.

From the data presented in Figure 17 and Figure 18 and Table 5 and Table 6, it is evident that the average relative error and the quality of the reconstructed flame temperature field using asymmetric light paths are inferior compared to those using symmetric light paths. As a result, achieving high-quality reconstruction of the flow field along the dominant light path is of primary concern. Following optimization using the Harris’s Hawk Optimization (HHO) algorithm, the reconstruction error of the asymmetric stochastic light path is reduced significantly on the dominant side, with larger errors typically concentrated on the weaker side. The number and configuration of light path arrangements have a notable impact on the final error magnitude. Compared to the simulated annealing algorithm, the HHO algorithm demonstrates superior optimization results for asymmetric light paths. This advantage stems from HHO’s ability to perform quicker perturbations in each iteration compared to simulated annealing. Moreover, HHO achieves a higher fitness level in generating asymmetric light paths, requiring less computational time than simulated annealing. Therefore, for optimizing asymmetric light paths, HHO proves to be a more suitable and efficient choice.

**Figure 19:**
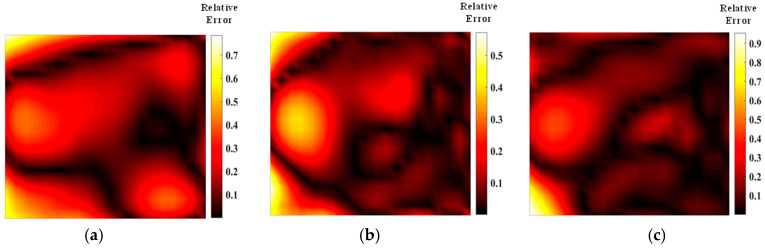
Mean error distribution of concentration in SA-optimized asymmetric optical paths: (**a**) 20 beams; (**b**) 32 beams; (**c**) 40 beams.

**Figure 20:**
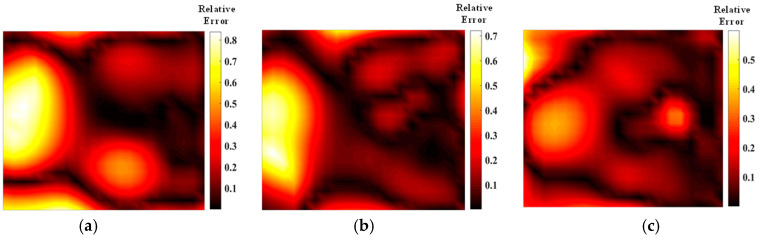
HHO-optimized asymmetric optical path concentration mean error distribution: (**a**) 20 beams; (**b**) 32 beams; (**c**) 40 beams.

Figure 19 and Figure 20 show the concentration field error distribution of the asymmetric flame reconstructed by the two optimization algorithms. Table 6 shows the error of HHO algorithm is smaller than that of SA algorithm. Based on the concentration measurement results, it is evident that the use of an asymmetric optical path for concentration measurement leads to significant error. Therefore, whenever applying an asymmetric optical path for flow field measurement, it is advisable to minimize its usage for concentration measurement. For the two optimization algorithms, the number of beams yielding the smallest average error in measuring the flame concentration field is 32 and 40, respectively. In the case of asymmetric optical path arrangements, the sparsity of the path arrangement greatly influences the reconstruction results. During subsequent optimization, emphasis should be placed on achieving a more uniform distribution of the optical path within the measurement area. Additionally, special attention should be given to adjusting parameters in the optical path generation process to ensure that weaker measurements on the weaker side of the optical path contribute meaningfully to the overall measurements. Further research is warranted to delve into this aspect.

## 4. Conclusions

In this paper, we developed a model for optimizing stochastic light paths based on the measurement of flame temperature and concentration fields using the Tunable Diode Laser Absorption Spectroscopy (TDLAS) technique. The optimization model was established using both the simulated annealing algorithm and the Harris’s Hawk Optimization (HHO) algorithm, specifically targeting non-axisymmetric flames. The main conclusions are as follows:

(1) The detection model based on the TDLAS technique demonstrates effective reconstruction for both asymmetric flame temperature and concentration fields. Under 40 parallel light paths, the relative error of temperature field reconstruction is 5.1%, and the relative error of concentration field reconstruction is 8.3%. The model provides high imaging accuracy and quality, validating its correctness.
(2) Both the simulated annealing and Harris’s Hawk algorithms were employed to optimize the light path arrangement for configurations of 20, 32, and 40 beams. Results indicate that the optimized optical path arrangements significantly outperform conventional regular arrangements. Moreover, the HHO algorithm surpasses the simulated annealing algorithm in terms of both optimization results and computational efficiency. Compared with the parallel optical path, the optimized optical path of the HHO algorithm reduces the temperature field error by 25.5% and the concentration field error by 26.5%.
(3) The optimization results for the asymmetric measurement optical path show that measurements on the dominant side of the optical path generation are reliable. However, the concentration measurement error is notably larger than the temperature measurement error.

This research is applicable to the field of non-contact sensing where optical path layouts or acoustic wave distributions are required [22,23,24]. In subsequent studies, we will focus on exploring the deeper mathematical coupling effects between randomized optimization of optical path layouts and physical information. This effort aims to establish a more comprehensive theoretical foundation.

## Figures and Tables

**Table 1:** Parameter settings for simulated annealing algorithm.

Initial Temperature	Closing Temperature	Cooling Rate	Chain Length
$T_{0}$	$T_{\mathrm{end}}$	*q*	*L*
100	1	0.9	10

**Table 2:** Mean relative error of flame temperature concentration field after SA optimization.

Error Level	20 Beams	32 Beams	40 Beams
Mean relative error of temperature	0.069	0.050	0.044
Relative error of mean concentration	0.105	0.080	0.082

**Table 3:** Comparison of optimization effect between the SA algorithm and HHO algorithm.

Mean Relative Error	SA (20)	HHO (20)	SA (32)	HHO (32)	SA (40)	HHO (40)
temperature	0.069	0.049	0.051	0.031	0.044	0.038
concentration	0.105	0.112	0.080	0.064	0.081	0.061

**Table 4:** Comparison of computation time between the SA algorithm and HHO algorithm.

Algorithm	SA (20)	HHO (20)	SA (32)	HHO (32)	SA (40)	HHO (40)
runtime/h	4.393	4.270	4.590	3.967	4.672	4.546

**Table 5:** Flame temperature field errors after optimization of the two algorithms.

Average Error Level	20 Beams	32 Beams	40 Beams
SA	0.103	0.069	0.071
HHO	0.099	0.068	0.069

**Table 6:** Flame concentration field errors after optimization of the two algorithms.

Average Error Level	20 Beams	32 Beams	40 Beams
SA	0.248	0.171	0.134
HHO	0.190	0.117	0.124

## Data Availability

The raw data supporting the conclusions of this article will be made available by the authors on request.
